# Swine Small Intestine Sealing Performed by Different Vessel Sealing Devices: Ex-Vivo Test

**DOI:** 10.3390/vetsci8020034

**Published:** 2021-02-22

**Authors:** Luca Lacitignola, Annarita Imperante, Rodrigo Trisciuzzi, Nicola Zizzo, Alberto Maria Crovace, Francesco Staffieri

**Affiliations:** 1 Dipartimento dell’Emergenze e Trapianti di Organi (D.E.T.O.), Sezione di Cliniche Veterinarie e Produzioni Animali, Università degli Studi di Bari “Aldo Moro”, 70124 Bari, Italy; francesco.staffieri@uniba.it; 2 Dottorato di Ricerca in “Trapianti di Tessuti ed Organi e Terapie Cellulari”, Dipartimento dell’Emergenza e Trapianti di Organi (D.E.T.O.), Università degli Studi di Bari “Aldo Moro”, 70124 Bari, Italy; annarita.imperante@uniba.it (A.I.); rodrigo.trisciuzzi@uniba.it (R.T.); 3 Dipartimento di Medicina Veterinaria, Sez. di Anatomia Patologica, Università degli Studi di Bari “Aldo Moro”, 70010 Bari, Italy; nicola.zizzo@uniba.it; 4 Dipartimento di Scienze Mediche di Base, Neuroscienze e Organi di Senso, Università degli Studi di Bari “Aldo Moro”, 70124 Bari, Italy; alberto.crovace@uniba.it

**Keywords:** small intestine, vessel sealing device, radiofrequency vessel sealing device, harmonic scalpel, full-thickness biopsy, burst pressure, leak pressure, swine

## Abstract

This study aimed to evaluate the sealing quality of swine small intestine using different laparoscopic radiofrequency vessel sealing devices (two 5 mm: RFVS-1 and -2; one 10 mm: RFVS-3) and a harmonic scalpel (HS) compared to golden standard closure technique. The study was divided into two arms. In study arm 1: n = 50 swine intestinal loops (10 per group) were transected with each instrument and the loops in which the devices provided complete sealing, at the gross inspection, were tested for maximum burst pressure (BP) and histological evaluation and compared to an automatic linear stapler. After the BP tests, the devices that achieved significantly lower BP values were excluded from the second arm. The RFVS-1 and -3 provided statistically significant results and were used in study arm 2, to obtain full-thickness biopsies along the antimesenteric border of the loop and were compared with hand-sewn intestinal closure (n = 30; 10 per group). The biopsies were histologically evaluated for thermal injury and diagnostic features, and intestinal loops tested for BP. RFVS-3 achieved comparable results (69.78 ± 4.23 mmHg, interquartile range (IQR) 5.8) to stapler closing technique (71.09 ± 4.22 mmHg, IQR 4.38; *p* > 0.05), while the RFVS-1 resulted in significantly (*p* < 0.05) lower BP (45.28 ± 15.23 mmHg, IQR 24.95) but over the physiological range, conversely to RFVS-2 (20.16 ± 7.19 mmHg, IQR 12.02) and HS (not measurable). RFVS-3 resulted not significantly different (*p* > 0.05) (45.09 ± 8.75 mmHg, IQR 10.48) than Suture (35.71 ± 17.51 mmHg, IQR 23.77); RFVS-1 resulted significantly lower values (23.96 ± 10.63 mmHg, IQR 9.62; *p* < 0.05). All biopsies were judged diagnostic. Data confirmed that RFVS-1 and -3 devices provided suitable intestinal sealing, with BP pressures over the physiological range. Conversely, the HS and RFVS-2 should not be considered for intestinal sealing. RFVS devices could be employed to obtain small intestine stump closure or full-thickness biopsies. However, further studies should be performed in live animals to assess the role of the healing process.

## 1. Introduction

Vessel-sealing devices (VSD) are routinely used in open and laparoscopic surgical procedures to provide hemostasis and to dissect tissue structures and blood vessels [1].

Radiofrequency (RF)-powered devices, such as those controlled by an impedance-based closed-loop feedback system, apply a combination of energy and mechanical pressure to cause a physical denaturation and reconfiguration of cellular proteins, particularly collagen and elastin, which upon cooling form a stable, leak-resistant, and hemostatic tissue plug. RF and harmonic scalpel (HS) systems are capable of permanently sealing blood vessels up to 5–7 mm in diameter, resulting in mechanical strengths comparable to or even more significant than that achieved by other mechanical devices as sutures, staples, and clips [2].

Off-label indications of energy-based devices were recently reviewed [3]. Studies performed on human patients were additionally found to predominantly use bipolar RF technology for parenchymal and ductal occlusion. A similar trend was seen among animal studies with only a handful of papers examining bipolar RF energy use for intestinal anastomosis. Although sutures and staples are the gold standard methods of intestinal sealing, intestinal thermofusion has been described as an alternative method for intestinal fusion and is used to seal intestines for transection [2,4,5,6,7,8] or anastomosis [2,9,10,11,12,13,14,15,16,17]. However, there are significant incongruent data about the maximum burst pressure measurements provided by the thermofusion. To our knowledge, no study has been performed evaluating the application of vessel sealing devices to obtain full-thickness intestinal biopsies.

In the veterinary scenario, the peritoneal cavity contamination and technically demanding tasks (i.e., intra-abdominal suturing and knotting), during intestinal surgical procedures, including intestinal biopsies, could limit a total laparoscopic approach. To obviate to this complication, it has been described the laparoscopic-assisted procedures, in which intestine manipulation, enterotomies, anastomosis, and full-thickness biopsies were managed outside the abdominal cavity [18,19]. However, evaluating the possibility of using total laparoscopic procedures in performing intestinal stumps or full-thickness biopsies should have the advantage of minimizing surgical trauma while maintaining a minimally invasive environment. Given the native use of radiofrequency vessel-sealing (RFVS) and HS also for laparoscopic procedures, we decided to carry out a preliminary ex-vivo study in a porcine model. The use of this model was justified by previous translational studies [20], and for the wide availability, reduced costs, and reasonable ethical issues. For these reasons, this study aimed, firstly, to evaluate the quality of sealing in swine small intestine transection provided by different RF and HS vessel-sealing devices compared to endoscopic stapler device. Next, we assessed the feasibility of full-thickness small intestinal biopsies performed by vessel sealing devices that provided significant results in the first part of the study. Based on the reported studies, we hypothesized that radiofrequency vessel-sealing (RFVS) devices provide sealing of the small intestine comparable to gold standard technique that they can be used to obtain safe and diagnostic intestinal biopsies.

## 2. Materials and Methods 

### 2.1. Samples

Eighty (n = 80) jejunal samples, 20 cm long (diameter ~ 2 cm, thickness ~3 mm), were harvested from four healthy female large white pigs, weighing 60 kg from slaughterhouse. Because specimens were obtained from slaughtered animals, no approval was needed from the Ethical Committee, according to the national law. 

After the harvesting, the jejunum was sampled and stored in saline solution and transported in a refrigerated box (4 °C) at Section of Veterinary Clinics and Animal Production, University of Bari, Italy. Then, the samples were stored at room temperature for 60 min. Experiments were performed ~90 min after harvesting. The samples were randomly assigned to eight different groups of 10 each, according to a randomization list obtained from a website (www.randomization.com).

### 2.2. Study Design

**Figure 1:**
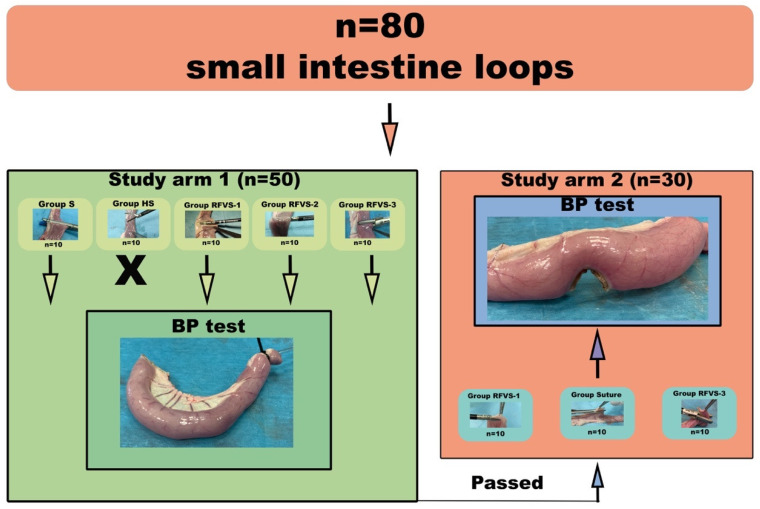
Study design. Eighty swine intestinal loops were randomly assigned to each experimental group (n = 10 each). Group S, Stapler; Group HS, Harmonic Scalpel; Group RFVS-1, LigaSure 5-mm Dolphin tip; Group RFVS-2, ENSEAL; Group RFVS-3, LigaSure Atlas 10-mm; BP, Burst Pressure. Group HS was not tested for maximum BP because it failed closure in all specimens. Devices provided adequate sealing in study arm 1 passed to study arm 2.

The study had two arms. In the first arm (study arm 1), fifty (n = 50) specimens were transected with five different techniques along the short axis. The loops in which the devices provided complete sealing, at the gross inspection, were tested for BP test. After the BP tests, the devices that achieved significantly lower BP values were not employed for the second arm study. In the second arm (study arm 2), thirty (n = 30) full-thickness biopsies were obtained from each specimen along the longitudinal axis of the loop with three techniques (Figure 1).

### 2.3. Experimental Groups of Study Arm 1

**Figure 2:**
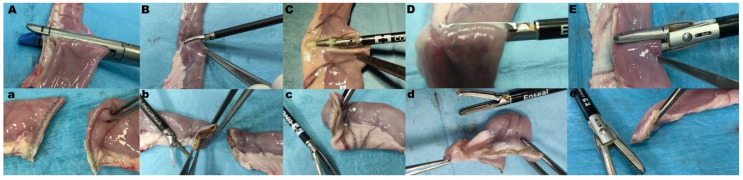
Representative pictures of device application for transection in study arm 1. Top row, during application; bottom row, after application. (**A**,**a**) Group S; (**B**,**b**) Group HS; (**C**,**c**) Group RFVS-1; (**D**,**d**) Group RFVS-2; (**E**,**e**) Group RFVS-3.

In Group S (n = 10), a 45 mm endoscopic stapler (Endopath^®^ ETS 45mm Articulating Linear Cutter, ref. ATS45, Ethicon Endo-Surgery Inc., Cincinnati, OH., USA) was employed with blue cartridge (3.5 mm titanium staples; Endopath ETS45 3.5MM, ref. TR45B, Ethicon Endo-Surgery Inc., Cincinnati, OH, USA). In group HS (n = 10), a 5 mm laparoscopic harmonic scalpel (Harmonic ACE+, ref. HAR36 Ethicon Endo-Surgery Inc., Cincinnati, OH, USA) was used for jejunum transection. The power setting was level 5, which was applied until complete transection was obtained. In group RFVS-1 (n = 10), transection was obtained with a 5 mm radiofrequency vessel-sealing device (LigaSure™ Dolphin Tip Laparoscopic Sealer/Divider, 37 cm, ref. LS1500, Medtronic Italia S.p.A., Milan, Italy) with straight jaws. In the group RFVS-2 (n = 10), the jejunum was transected with a laparoscopic 5 mm radiofrequency vessel sealing device with curved jaws (Enseal^®^ Trio Tissue Sealers, ref. ETRIO335H, Ethicon Endo-Surgery Inc., Cincinnati, OH, USA). The instruments were connected to the same dedicated generator as the HS instrument (Ethicon Gen11 Generator, ref. Gen11, Ethicon Endo-Surgery Inc., Cincinnati, OH, USA). In the group RFVS-3 (n = 10) the transection was performed with a 10 mm radiofrequency vessel sealing device with straight jaws (LigaSure Atlas™ Tissue Fusion Laparoscopic Instrument, 37 cm, ref. LS1037, Medtronic Italia S.p.A., Milan, Italy). The instruments used in the RFVS-1 and -3 groups were connected to the same generator (ForceTriad™, Medtronic Italia S.p.A., Milan, Italy) with a power setting at 3 bars. The number of times the instruments were applied to achieve sealing and gross evaluation of sealing was recorded (Figure 2).

### 2.4. Sample Constructs

**Figure 3:**
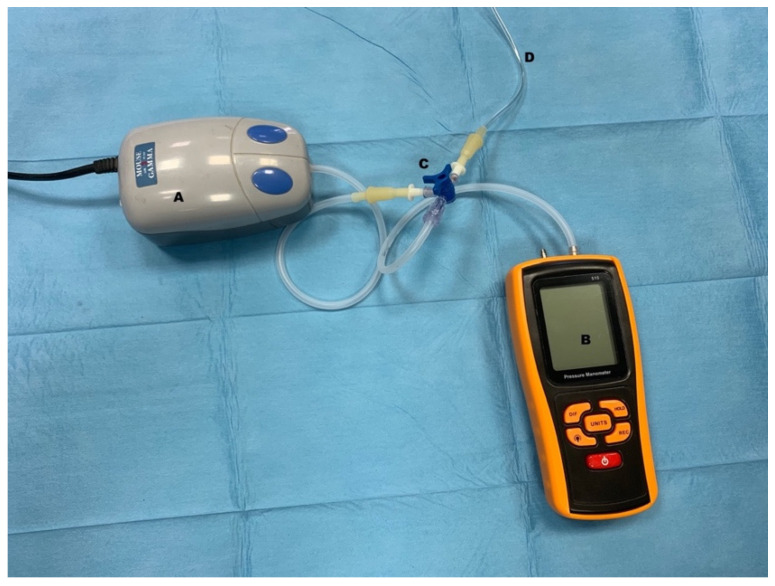
Image of instrumentation used for burst pressure (BP) test. (**A**) air pump; (**B**) manometer; (**C**) three-way stopcock; (**D**) tube to the construct.

Once the transection were performed, each specimen was stored in different boxes containing saline solution and stored at room temperature for the time need to complete the tests, and not exceeding 90 min. Accordingly, to the previous randomization list, a specimen was selected for setting up the construct. Each construct consisted of connecting an infusion set line inside the jejunal loop lumen for air administration. Then, a 4 mm polyethylene cable tie was tightened on the tube line, taking care not to let air leak around the tube. The air infusion line was connected to a three-way stopcock. An air pump (Airpump M103, Croci s.p.a, Varese, Italy) was used to inflate the construct with a flow rate of 2.5 L/min plugged to the stopcock. On the other plug of the stopcock, a digital manometer (GM510, RGBS, Guangdong, China) was connected for continuous and maximum pressure recording (Figure 3).

### 2.5. Burst and Leak Pressures

The burst and leak pressures of each method were measured using the pressure manometry. The air leaking was observed dipping the sealed site under water to see bubbles. Maximum pressure was automatically recorded by the digital manometer when air leaking or complete failing (burst) of sealed intestine occurred, and the manometer recorded a pressure decrease. Maximum pressure in mmHg, air leaking, or failure of the construct was recorded.

### 2.6. Experimental Groups of Study Arm 2

In this part of the study, the devices that passed the sealing during the BP evaluation were used to obtain full-thickness biopsies on the antimesenteric border of the intestinal loops, and they were compared to full-thickness incisional biopsies. To obtain full-thickness biopsies, the device jaws were applied on the antimesenteric border along the longitudinal axis of the intestinal loop with the help of surgical forceps. Then, the instrument jaws were clamped, and energy was activated at level 3 (3 bars) on the generator. Surgical incision biopsies were obtained from the antimesenteric border of the intestinal loop with Metzenbaum scissors (cold blade), and the wound was closed with seven sutures of Gambee’s interrupted pattern performed with USP 4-0 polydioxanone suture with 13 mm ½ circle needle (PDS*II, Z924, Ethicon, Cincinnati, OH, USA).

**Figure 4:**
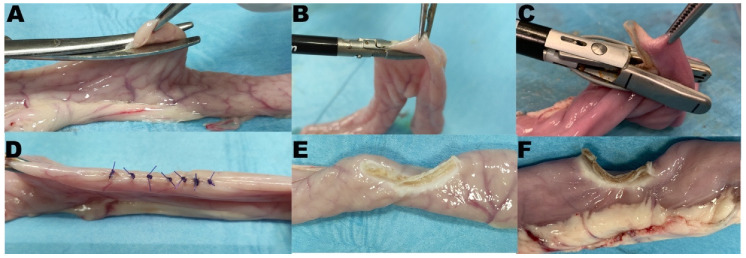
Representative pictures of biopsy procedure in study arm 2. Top row, during application; bottom row, after biopsy. (**A**,**D**) Group Suture; (**B**,**E**) Group radiofrequency vessel-sealing (RFVS)-1; (**C**,**F**) Group RFVS-3.

The instrument activation and biopsy length numbers were recorded (Figure 4.)

The specimens were prepared as described for the study arm, applying another cable tie at the opposite end to firmly close the specimen. After proper construct assembly, they were tested for burst and leak pressures as described previously.

### 2.7. Histology

Tissues obtained from the transected intestinal tract (study arm 1) and biopsies (study arm 2) were fixed in 10% neutral-buffered formalin at the time of initial collection, routinely processed, embedded in paraffin, cut at 3–5 mm, and stained with hematoxylin and eosin. For the histological evaluation, in study arm 1, in order to avoid the influence of pressure tests on the sealed site, after performing the transection with each instrument, a portion of the divided loop was destined for the BP test, while the opposite part for the histology. The surgical biopsy specimens from study arm 2 were categorized as acceptable or unacceptable diagnostic quality by a pathologist blinded to the procedure. For this study, the diagnostic quality was defined according to a previous study [21], briefly, as a specimen in which ≤25% of the total section area was distorted/destroyed as an artifact of the biopsy technique. 

### 2.8. Statistical Analysis

Statistical analysis was performed with available software (Minitab^®^ 19 Statistical Software, Suturentry, UK). Data were assessed for normality of distribution with the Shapiro–Wilk test. Data were reported as the mean ± SD, and interquartile range (IQR). A 1-way ANOVA was used to compare results among groups. *p*-values < 0.05 were considered significant. Power analysis was performed by a specific analysis software (G*Power 3.1, Statistical Power Analyses, Dusseldorf, Germany). The sample size was calculated on the basis of a pilot study considering an effect size of 2.70, alfa 0.05, and power of 95%. The calculated sample size “a priori” for 1-way ANOVA test was n = 8. Thus, we included in the study 10 samples in each experimental group.

## 3. Results

### 3.1. Study Arm 1

In group HS, the instrument was activated to complete the transection for a mean of 3.4 ± 0.516 times, (IQR 1); in the group RFVS-1, the mean was 4.3 ± 0.483 (IQR 1); with RFVS-2, the mean was 2.9 ± 0.316 (IQR 0); with RFVS-3, the mean was 2.3 ± 0.483 times (IQR 1); with S, the mean was 1 ± 0 (IQR 0). 

At gross evaluation, the sealing succeeded in 100% of samples in groups S, RFVS-1, and RFVS-3, and 80% in RFVS-2. In the group HS, 100% of samples failed to seal, producing a sample not evaluable for pressure testing. Thus, samples from this group were eliminated from the study.

**Figure 5:**
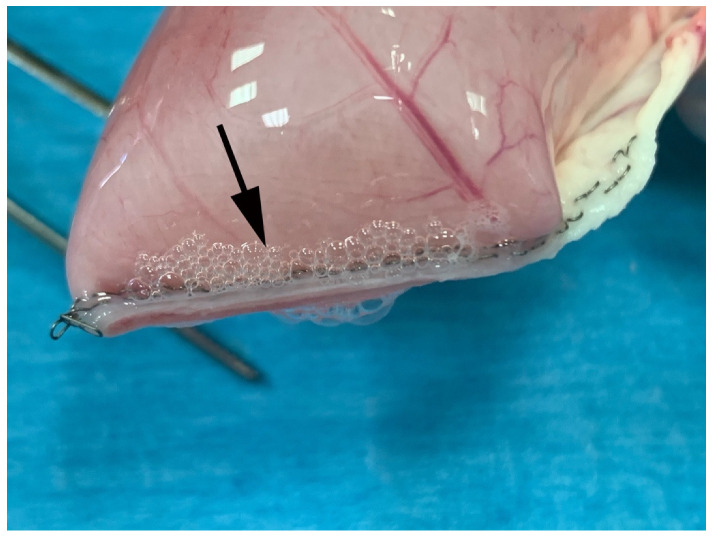
Representative image of Group S specimen during BP test. Air leaking was found at the staples site (black arrow). No failure of the construct was noted.

Maximum burst or leak pressure data are reported in Table 1. In the stapler group (S), none of the constructs failed, but air leaking was constant at the staples (Figure 5).

**Figure 6:**
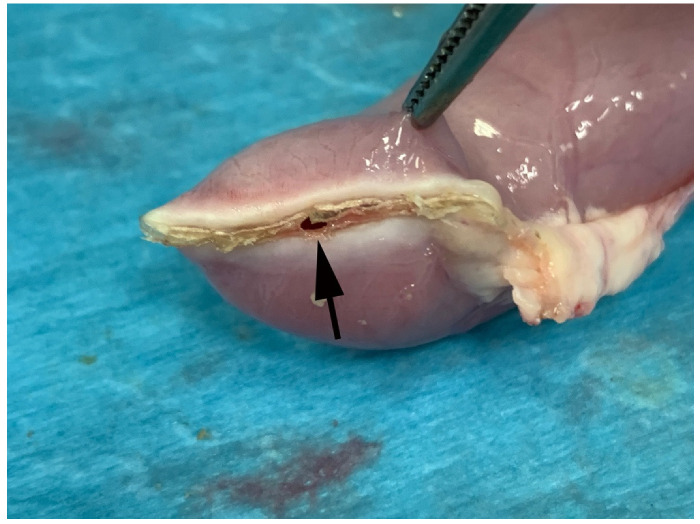
Representative image of RFVS-3 group specimen during BP test. Note sealing failure (black arrow).

The maximum pressure detected before leaking did not significantly differ (*p* > 0.05) from that of group RFVS-3. Contrarily to Group S, all constructs burst at the maximum pressure, such as in RFVS-1 (Figure 6). 

**Figure 7:**
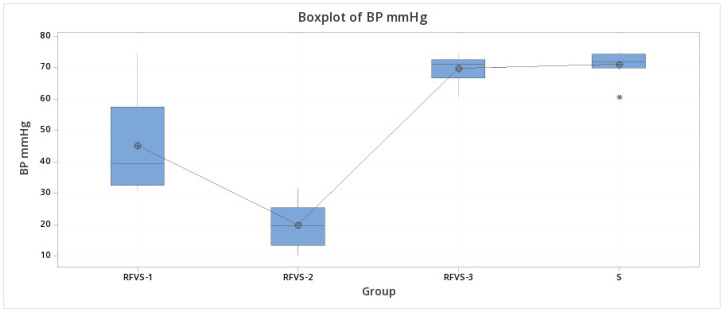
Boxplot of BP test. Mean maximum BP values for each group in Study arm 1. Group S, Stapler; Group RFVS-1, LigaSure 5 mm Dolphin tip; Group RFVS-2, ENSEAL; Group RFVS-3, LigaSure Atlas 10 mm; BP, Burst Pressure). Group HS was not tested because of sealing failure in all samples. RFVS-1 was significantly lower (*p* < 0.05) than the RFVS-3 and S groups, but significantly higher than the group RFVS-2, which recorded the significantly lowest values (*p* < 0.05). *: outlier.

Maximum burst pressure in the RFVS-1 was significantly lower (*p* < 0.05) than the RFVS-3 and group S, but significantly higher than the RFVS-2 group, which recorded the significantly lowest values (*p* < 0.05). Figure 7 shows the related plot of BP. 

### 3.2. Study Arm 2

In this part of the study, only RFVS-1 and RFVS-3 were employed in the experiment because they did not fail in the previous arm of the study.

**Figure 8:**
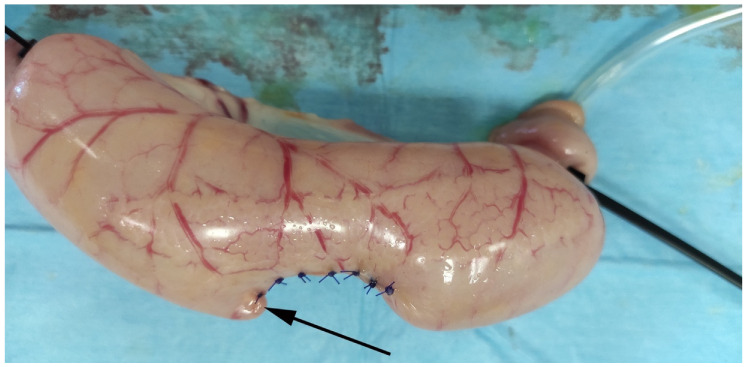
Representative image of the group suture construct during the BP test in study arm 2. The black arrow shows the failure site.

In both RFVS-1 and -3, the instruments were activated two times to complete the biopsy. In the Suture group, air leaking was observed at maximum pressure (Figure 8).

**Figure 9:**
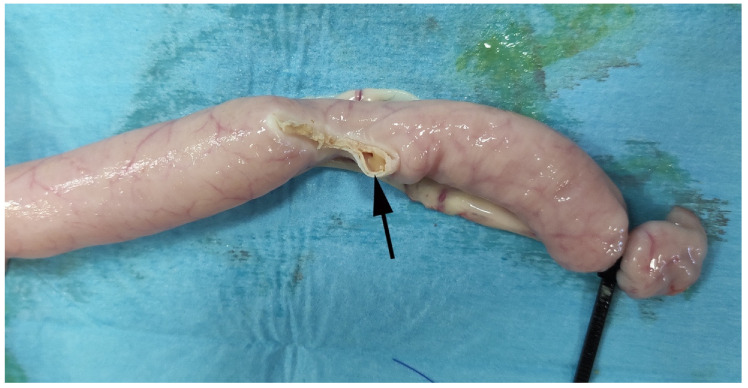
Representative image of RFVS-3 construct during BP test in study arm 2. The black arrow shows the failure site at maximum pressure.

All specimens of groups RFVS-1 and RFVS-3 burst at maximum pressure (Figure 9).

**Figure 10:**
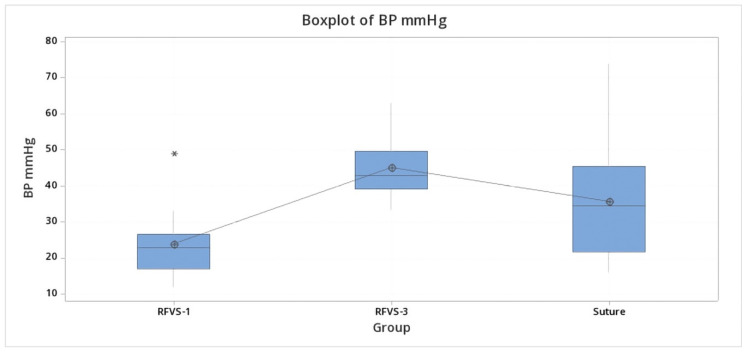
Boxplot of BP test. Mean maximum BP values for each group in Study arm 1. Group Suture; Group RFVS-1, LigaSure 5 mm Dolphin tip; Group RFVS-3, LigaSure Atlas 10 mm; BP, burst pressure). The group RFVS-1 resulted in significantly lower BP values (*p* < 0.05). Otherwise, RFVS-3 and the Suture group showed comparable BP pressures (*p* > 0.05). *: Outlier.

Table 2 summarizes the results of the burst pressure test. The group RFVS-1 resulted in significantly lower BP values (*p* < 0.05). Otherwise, RFVS-3 and Suture group showed comparable BP pressures (*p* > 0.05) (Figure 10).

Lengths of biopsies are summarized in Table 3. In the group RFVS-1, significantly smaller biopsies were obtained (*p* < 0.05). No significantly different biopsies sizes were detected between the RFVS-3 and Suture groups.

Maximum BP pressure of RFVS-1 and -3 obtained from the evaluation of both arms showed significantly lower values in biopsied constructs related to transection (*p* < 0.05) (Table 4).

### 3.3. Histology

**Figure 11:**
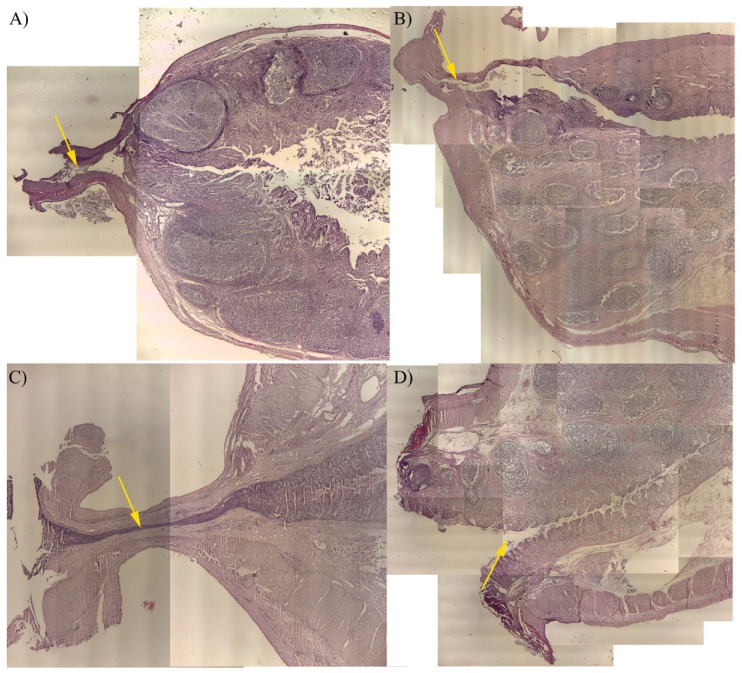
Representative histological images obtained merging different fields of view at 4× magnification (E.E.). Sealed edge from samples obtained in study arm 1. (**A**) RFVS-1; (**B**) RFVS-2; (**C**) RFVS-3; (**D**) HS. Yellow arrows show the sealed site. Poor sealing of all layers in RFVS-1 and -2. Complete sealing of all layers, including the mucosa obtained with RFVS-3. Incomplete sealing obtained with HS, resulting in complete inability to fuse the edge of the loop.

Intestine samples from study arm 1 were examined histologically to determine the efficacy of the seal (Figure 11). 

Analysis of samples closed with the stapler was not examined because of the presence of titanium staples, which made it impossible to perform adequate processing of the samples. Furthermore, we considered staple removal not suitable to assess the quality of closure. In group HS, the seals showed a lack of sealing, with complete separation of the tissues, resulting only in the cut edge of the samples. The thermal and mechanical injury was located nearby the closure site of all specimens obtained by RF devices. The tissue appeared compressed and markedly elongated with modified architecture. The mucosal architecture was obliterated, leaving a heat-induced coagulum with holes in the tissue and no appreciable cellular architecture.

In RFVS-1 group, the seal showed compression, but incomplete closure of the lumen and mucosal layer. Similar findings were noted in the RFVS-2 group. In the RFVS-3 group, the lumen was completely closed, with the mucosal and muscular layers lying close to one another. The mucosal layer appeared as a thin structure, suggesting the effectiveness of the compression applied and success of the seal. 

**Figure 12:**
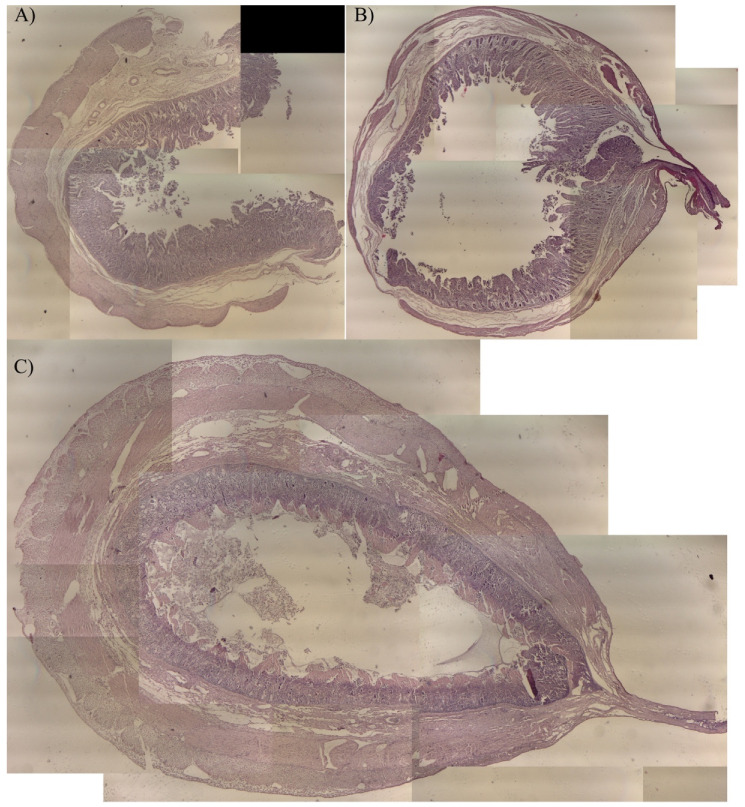
Representative histological images obtained merging different fields of view at 4× magnification (E.E.). Cross sectional slides from biopsies obtained in study arm 2. (**A**) Group Suture (biopsies obtained with cold blade); (**B**) RFVS-1; (**C**) RFVS-3.

All of the biopsy samples obtained with the cold blade in study arm 2 were fully diagnostic. Biopsies harvested in-group RFVS-1 and -3 had >25% of area section, which was considered diagnostic by the pathologist. The thermal and mechanical injury was located close to the cut side of the specimen, and in all specimens, the architecture of the remaining area was classified as normal and fully diagnostic (Figure 12).

## 4. Discussion

In this study, we evaluated the quality of sealing of swine small intestine transection provided by different RF and HS vessel sealing devices compared to the endoscopic stapler device. After that, we evaluated the feasibility of full-thickness small intestinal biopsies by vessel sealing devices that provided significant results in the first part of the study. Based on the reported studies, we hypothesized that RFVS devices provide sealing of the small intestine comparable to the gold standard techniques, and can be used to obtain safe and diagnostic intestinal biopsies.

The strength of this tissue connection probably depends on tissue parameters, such as protein content, tissue thickness, and perfusion, as well as process parameters, such as compressive pressure, fusion temperature, and duration of heating [17]. Bipolar radiofrequency-induced thermofusion of intestinal tissue is based on a denaturing process of collagen type I, elastin, and further collagens. While heating the tissue, the triple helical structure of collagen type I monomers uncoils and transmutes into a random coiled mass of peptide chains [22].

At a gross evaluation, the sealing succeeded in 100% of samples in Group S, RFVS-1, and RFVS-3; 80% in RFVS-2. In Group HS, 100% of samples failed to seal, producing a sample that was not evaluable for pressure testing. Thus, samples from this group were eliminated from the study. This finding was in contrast with the results obtained comparing HS device with RFVS in tissue dissection and vessel sealing. In fact, it has been reported that for the native indications as a vessel sealer and dissector, the HS showed similar results to the RFVS devices [23]. We suppose that the different content in term of elastin and collagen of the intestine compared to vessels could dramatically influence the HS ability to intestine sealing.

The results of the first arm showed that in the HS group, the instrument had been activated a mean of 3.4 times to complete the transection. In the group RFVS-1, the mean was 4.3; with RFVS-2, the meanly was 2.9; with RFVS-3, the mean was 2.3′ and with S, the mean was 1. In RFVS devices, only one cycle per bite was performed, and each activation was applied after the previous one to complete the transection along the short axis of the intestine. It has been described that the power level and the number of cycles applied at the same bite (without advancing the jaws) can affect the maximum BP [4]. Thus, for this reason, we decided to employ the maximum power level with a single activation. In any case, the exact energy delivered cannot be calculated, because all RFVS devices employed in this study had a feedback-controlled energy adjustment, which measures the tissue impedance starting from the closure of the device and 200 times per second over the whole sealing cycle. Based on these measurements, an algorithm within the generator adjusted the output based on the changes of the tissue impedance. The output of the generator can vary within the sealing cycle between 0 and 150 W [9]. No control of the cycle time is obviously under the direct control of the surgeon. In contrast, HS devices sequentially convert electrical energy into mechanical energy and then into thermal energy to facilitate tissue sealing, but without the passage of electrical current through the tissue [24]. Thus, with HS devices, the cycle time is directly under the surgeon’s control.

The use of smaller devices inevitably needs to be activated several times to complete the transection, and obviously, some sealed tissue should be overlapped by consequent activation. The length of the instrument’s jaws could also afflict the tissue sealing. In our study, we employed devices that had a cut length from 12 mm in RFVS-1, 15 mm in RFVS-2, 22 mm in RFVS-3, and 14.2 mm in the HS device, while the stapler had a length of 45 mm. Thus, the use of instruments of adequate cutting length could prevent double or more activation on the same portion of tissue, avoiding excessive thermal effects on tissues providing negative effects on the quality of sealing [4].

The compressive pressure deployed by the instrument’s jaws has been demonstrated to influence the tissue sealing [2,17,22,25,26,27]. It has been established that the optimal compression for the small intestine was in the 0.15–0.25 MPa range [3]. RFVS and HS devices tested in this study had different compression pressure mechanisms, which were assessed by the surgeon in HS and RFVS-2 devices. On the contrary, for RFVS-1 and -3, as well as the stapler, the compression is controlled by the automatic clamping lock in the instruments’ handle. Moreover, compression pressure has been shown to be not uniform along the instruments’ jaws. Indeed, appropriate compression has been applied at the base of the jaws and gradually diminished on the tip [25,27]. This phenomenon could explain different sealing abilities among devices. 

Although no direct temperature measurement was performed in this study among the different devices, the thermal effects on the small intestine should be considered. The RF devices were observed to generate a much lower tip temperature on activation when compared to the HS (45.8 vs. 172.6 °C) [3,28]. It is likely that multiple factors, such as thermal injury, the compression pressure not being controlled, and different sealing mechanisms, negatively affected the small intestine sealing provided by the HS instrument, resulting in complete failure of sealing in our study. For this reason, we excluded the HS group from BP evaluation.

Maximum burst or leak pressure was performed in RFVS devices compared to the stapler in study arm 1. In our study, maximum BP results for RFVS-1 were 45.28 mmHg, for RFVS-2 were 20.16 mmHg, for RFVS-3 were 69.78 mmHg, and for S were 71.09 mmHg. In the group S, no constructs failed completely, but constant air leaking was noted at the staples level. The maximum pressure detected before air leaking did not significantly differ (*p* > 0.05) from the RFVS-3 group. Contrarily to the group S, all constructs burst at maximum pressure, such as with RFVS-1. Maximum burst pressure in RFVS-1 was significantly lower (*p* < 0.05) than in the RFVS-3 and S groups, but significantly higher than in the RFVS-2 group, which recorded the significantly weakest values (*p* < 0.05). Our results were in line with most published data. In fact, the maximum BP described ranged from 39.8 to 60.28 mmHg [2,4,9].

In study arm 2, we evaluated the feasibility of obtaining small intestinal diagnostic biopsies using RFVS devices compared to surgical biopsies collected with a cold blade for intestinal wounds closed with a hand-sewn technique (Gambee’s interrupted suture pattern). 

To obtain an adequate biopsy sample, RFVS devices were activated two times to complete the cut. 

The group RFVS-1 resulted in significantly lower BP values (23.96 mmHg; *p* < 0.05). Otherwise, RFVS-3 (45.09 mmHg) and Suture group (35.71 mmHg) showed comparable BP pressures (*p* > 0.05). Interestingly, these values were significantly lower than maximum BP pressure obtained in study arm 1 (*p* < 0.05). We suppose that the reduction in intestinal diameter for obtaining a full-thickness biopsy dramatically reduced the BP compared to the intestinal transection where the full intestinal diameter was maintained. This phenomenon can be explained with Laplace’s law, according to which the wall of a distensible organ is in balance if the distending force (intraluminal pressure) is balanced by the wall tension (partly passive, the elasticity of the organ; partly active, generated by the contraction of smooth muscle). The wall of the organ breaks if the developed wall tension is not sufficient to support intraluminal pressure. If the radius of the intestine decreases, the wall tension dramatically decreases, and intraluminal pressure breaks the organ at the sealing line (locus minoris resistentiae). Similar to other hollow organs, such as vessels, in a long pliable tube, the site of the largest diameter requires the least pressure to distend [14,29]. 

Another explanation could be related to the direction of the biopsy. In fact, the tension lines on the wall of a hollow organ propagate perpendicular to the cutting line (hoop stress), exerting a direct force. On the contrary, if the cutting line is carried out transversely, the tension forces are parallel and are more influenced by longitudinal stress. Considering the tested construct as a thin wall cylindrical tank, the hoop stress is equal to twice the longitudinal stress. The cylindrical pressure would split on the wall instead of being pulled apart (as it would under an axial load). In one study, the effect of the direction of small intestine biopsies was studied [30]. The results of this study have shown that there is no difference between the two directions, but the test performed was carried out only at pressures of 15 and 25 mmHg, lacking the maximum BP values.

In un-anesthetized dogs, the mean resting pressure was reported as 6.8 ± 0.77 mmHg in the proximal jejunum, 6.4 ± 0.63 mmHg in the middle jejunum, and 6.7 ± 0.54 mmHg in the distal jejunum [31]. In human beings, during intestinal pressure waves, the pressure may rise to maximum levels of 50 mmHg in the physiological setting [9,32,33,34], while in dogs, the physiologic intraluminal pressures reach 25 mmHg during peristalsis [35]. Dogs with experimentally induced complete ileal obstruction had a maximum intraluminal pressure of 44 mm Hg at 3 days after obstruction [36]. It has been shown that the intestine motility is strongly reduced after intestinal surgery and the mean pressure decreased to 6.1 ± 5.8 mmHg during anesthesia [37]. In our study, maximum BP of the sealed small intestine with RFVS-1 and -3 resulted in similar or even twofold higher values than previously reported physiological data, as well as a stapler or hand-sewn closure.

Histological results of both study arms confirmed and strengthened the biomechanical findings. Indeed, in study arm 1, the superiority of the RFVS-3 device was detected in terms of quality of sealing when compared with the other devices employed in this study. In particular, in the HS and RFVS-2 group, quality of sealing was considered not suitable and poor, respectively. Adequate sealing, although lower than RFVS-3, has been appreciated for the 5 mm device used in the RFVS-1 group. Although these two devices are based on the same RF technology, the different force of clamping and power delivery could have afflicted the sealing quality. In this study, we have not performed a quantitative measuring of the extension of the thermal and mechanical injury, in the stumps obtained in study arm 1, limiting the evaluation to a qualitative analysis. However, it has been reported that the thermal spread during the use of RF and HS devices varied in function of the instruments employed, time of activation, the pressure of jaws on the tissue, and type of tissue sealed [3]. In fact, in a study performed on muscles for the evaluation of lateral thermal spread, RFVS showed a rise of 25 °C at 1 mm from the device and were < 1.0 °C at 5 and 10 mm [38]. In another study performed on artery segments, the RFVS showed lateral thermal spread at 3.8 mm and on vein segments at 3.4 mm [23]. In a study performed in a swine model of small intestine thermofusion the mean lateral destruction of the tissue structures on each side of the fusion line for RFVS device was 2.69 mm [2].

In study arm 2, as expected, the biopsies obtained with the cold blade were fully diagnostic. In this study, biopsies harvested with the RF devices, provided a thermal and mechanical injury located only at the sealing site. However, this artifact did not afflict most of the samples and was restricted only at 25% of cross-sectional area. Indeed, the small intestine architecture modification due to thermal and mechanical injury is impossible to avoid given the nature of current RF resection technologies, and should be considered as a limitation of RF devices. However, in this study, the diagnostic quality of samples obtained was considered suitable for pathologic diagnosis purposes, so the modification at the sealed site is expected with RF energy-based devices and surgeons should consider harvesting samples of proper size.

Our study had some limitations. Firstly, the burst and leak pressures for this study were directly determined after performing the transection or biopsy and the influence of healing, and therefore, increased stability of the stump or biopsied tissue could not be accounted for. Some studies have reported that the sealing strength of the early phase rises significantly through time [5,6]. In fact, anastomoses performed by RF devices were found to be macro- and microscopically intact seven days after surgery [5]. Elemen et al. reported increasing BP when tested on post-operative day 7 [39]. Recently, in a swine model of thermofused anastomosis [40], the percentage of intact anastomoses, after 2 weeks from surgery, was 73.3–93.3%, according to the different setting modes of the RFVS device applied. Moreover, the authors demonstrated that collagen fibers that were accumulated in the frame and filled in the gap between the two extremities of the muscle layer were considered as the main mechanical factors for the safety of anastomoses. Another study evaluated the survivable anastomotic resection of the small intestine using an RFVS device, resulting in immediately watertight and intact seals with undisturbed healing, complete with granulation tissue, newly synthesized collagen in the submucosa of joined intestine, and reepithelialization at seal borders. Furthermore, the treated animals displayed healthy and normal intestinal passage 7 days postoperatively [41]. In a study of cecal thermofusion with RFVS devices, the inflammatory cell infiltration, fibroblast growth, neoangiogenesis, and collagen disposition were similar to the closure obtained with mechanical staplers, but the ischemia levels were significantly higher in this group [6]. In a study performed on a rabbit model of RFVS thermofusion of the cecum, the authors found that the sealed tissue goes through an inflammatory process, progressively reabsorbing the necrotic material and developing a stromal reaction with the formation of connective tissue, which replaces the necrotic tissue [42]. However, the role of thermal spread and damage on the healing process after the intestinal thermofusion is still lacking strong evidence, and further experimental studies should be performed to ensure the safeness of the proposed technique, before suggesting the regular clinical use. 

Another limitation of the present study includes the non-physiologic method of testing and the use of cadaveric tissues that may hold sutures or behave differently from intestinal tissues in living animals. Testing of the fresh cadaver control tissues was performed to ensure that there had not been tissue breakdown and to enable the evaluation of the tissue strength relative to results. In fact, it has been demonstrated that fresh porcine jejunal segments may be used for determining intestinal leak pressure [43]. 

In study arm 2 we considered only the thermal effects, the quality of biopsies, and BP of the biopsied intestinal loop, lacking in the evaluation of the stricture created by the biopsy technique. We recognize it could represent a further limitation and we aim to perform a specific study on this issue.

## 5. Conclusions

In conclusion, based on our results, we can speculate that only some RFVS devices provide feasible sealing of the small intestine comparable to the stapler or hand-sewn closure. In particular, RFVS-3 (10 mm RFVS device) resulted in similar BP values to gold standard closure techniques either in small intestine closure or in harvesting full-thickness biopsies. While RFVS-1 (5 mm Dolphin tip RFVS device) achieved lower BP pressure, it ensured values higher than physiological parameters. We also obtained, diagnostically, biopsies that can easily be harvested without the need to suture. Because of the laparoscopic native use of these instruments, further studies using a total laparoscopic procedure should be investigated in vivo in an experimental animal model before vessel sealing devices can be used for intestinal closure in clinical cases.

## Figures and Tables

**Table 1:** BP test in study arm 1.

BP (mmHg)			
Group	Mean	St Dev	IQR
HS	*	*	*
RFVS-1	45.28	15.23	24.95
RFVS-2	20.16	7.19	12.02
RFVS-3	69.78	4.23	5.8
S	71.09	4.22	4.38

Group HS, Harmonic Scalpel; Group RFVS-1, LigaSure 5 mm Dolphin tip; Group RFVS-2, ENSEAL; Group RFVS-3, LigaSure Atlas 10 mm; Group S, Stapler; BP, burst pressure. St Dev: standard deviation. IQR: interquartile range. * missing data (BP test not performed).

**Table 2:** BP test in study arm 2.

BP mmHg			
Group	Mean	St Dev	IQR
RFVS-1	23.96	10.63	9.62
RFVS-3	45.09	8.75	10.48
Suture	35.71	17.51	23.77

Group Suture, intestinal wound closed with suture; Group RFVS-1, LigaSure 5 mm Dolphin tip; Group RFVS-3, LigaSure Atlas 10 mm; BP, burst pressure). St Dev: standard deviation. IQR: interquartile range.

**Table 3:** Biopsy length.

Biopsy Length (mm)			
Group	Mean	St Dev	IQR
RFVS-1	14.7	2.111	4.25
RFVS-3	21.5	1.65	3.25
Suture	23.1	4.04	5

Group Suture, intestinal wound closed with suture; Group RFVS-1, LigaSure 5 mm Dolphin tip; Group RFVS-3, LigaSure Atlas 10 mm; BP, burst length in mm). St Dev: standard deviation. IQR: interquartile range.

**Table 4:** Comparison of maximum BP pressure in mmHg obtained in the two-study arm.

BP (mmHg)			
	Mean	St Dev	IQR
RFVS-1			
Biopsy	23.96	10.63	9.62
Transection	45.28	15.23	24.95
RFVS-3			
Biopsy	45.09	8.75	10.48
Transection	69.78	4.23	5.8

RFVS-1, LigaSure 5 mm Dolphin tip; Group RFVS-3, LigaSure Atlas 10 mm; BP, burst pressure). St Dev: standard deviation. IQR: interquartile range.

## Data Availability

Data is contained within the article.
